# Porcine type I interferons: polymorphic sequences and activity against PRRSV

**DOI:** 10.1186/1753-6561-5-S4-S8

**Published:** 2011-06-03

**Authors:** Yongming Sang, Raymond RR Rowland, Frank Blecha

**Affiliations:** 1 Department of Anatomy and Physiology, College of Veterinary Medicine, Kansas State University, Manhattan, Kansas, USA; 2 Department of Diagnostic Medicine and Pathobiology, College of Veterinary Medicine, Kansas State University, Manhattan, Kansas, USA

## Abstract

**Background:**

Type I interferons (IFN) are a heterogeneous group of cytokines central to innate and adaptive antiviral immune responses. We have recently reported that porcine type I IFNs comprise at least 39 functional genes with diverse antiviral activity against porcine reproductive and respiratory syndrome virus (PRRSV). Here we report that potential cytokine polymorphisms exist in several genes of porcine type I IFNs.

**Results:**

We have detected more than 100 potential polymorphic mutations, which include nucleotide substitutions and deletions, within the coding regions of porcine type I IFNs. Approximately 50% of the nucleotide changes were mutations that resulted in non-conserved amino acid substitution, as well as deletions that produced frame shifts in the open reading frames (ORFs). We have identified more than 20 polymorphic mutants that showed alterations in anti-PRRSV and anti-vesicular stomatitis virus (VSV) activity *in vitro*. In particular, some mutations in *IFN-α2*, *IFN-α3*, *IFN-α8*, *IFN-α12* and *IFN-ω5* significantly altered the antiviral activity of expressed proteins in comparison to the wild-type or variant with more similarity to the wild-type.

**Conclusions:**

Multiple polymorphic isoforms potentially exist within subtypes of the porcine type I IFN family. Polymorphic mutations are more common in multiple-member subtypes than single-member subtypes, and most are found within the IFN-α subclass. Some polymorphic isoforms have altered amino acid composition and shifted ORFs, which show significantly different antiviral activity *in vitro*.

## Background

Type I interferons (IFNs) are a family of cytokines prominent in antiviral responses [1,2]. Genetic mutations that result in dysfunction of type I IFN production and actions are ultimately associated with susceptibility to viral diseases [2,3]. Conversely, viruses may antagonize or evade type I IFN actions resulting in successful infections [4]. In this context, porcine reproductive and respiratory syndrome virus (PRRSV), a RNA virus that causes devastating losses in the global swine industry, suppresses type I IFN production [5,6]; and diverse PRRSV strains manifest different abilities to suppress type I IFN expression [7]. Furthermore, emerging evidence implicates that cytokine polymorphisms such as those in IFN-γ and interleukin (IL)-8 are key factors in breed- or genotype-associated viral resistance [8-10]. However, how type I IFN polymorphisms affect resistance to PRRSV infection is not known. We have recently reported that the porcine type I IFN family consists of at least 39 functional genes, which are classified into seven subclasses of 17 IFN-α, 11 IFN-δ, and 7 IFN-ω subtypes, and single-subtype subclasses consisting of IFN-αω, IFN-β, IFN-ε, and IFN-κ [11]. We showed that members of different or the same porcine type I IFN subclasses have diverse expression profiles in various pig tissues and *in vitro* antiviral activities [11]. Here we report that expressed proteins from multiple polymorphic isoforms within subtypes of type I IFNs have significantly altered antiviral activity, which may represent an important factor for determining the outcome of the host-virus interaction.

## Methods

The isolation and analyses of IFN genes were conducted as previously described [11]. In brief, coding regions of porcine IFNs were amplified from several cDNA and genomic DNA pools using a high-fidelity PCR with subtype-common cloning primers. PCR fragments, which flank six nucleotides before the start codon and stop codon of each IFN ORF, were purified and cloned into a pcDNA™ 3.3-TOPO^®^ TA cloning/expression vector (Invitrogen, Carlsbad, CA) under the action of the vector CVM promoter. At least five clones for each IFN gene were sequenced and clones that showed the highest sequence identity (>97%) to the reference sequences (extracted from genomic draft sequences by Sanger Institute Porcine Project Team, which is available from http://www.ensembl.org/Sus_scrofa/index.html) were selected as polymorphic variants of a particular IFN for mutation definition and protein expression. Mutation determination was conducted by combined use of sequence analysis tools in Lasergene v8.1 (DNASTAR, Inc. Madison, WI) and Bioedit (http://www.mbio.ncsu.edu/BioEdit/bioedit.html). IFN peptides were collected and partially purified from supernatants of HEK293F cells transfected with individual IFN-expression plasmids. Authentic expression of individual IFN peptides was confirmed by gel electrophoresis; and peptide concentrations were adjusted to 2 μg/ml with the serum-free medium to ensure the antiviral differences were not a result of peptide levels in the antiviral assays (11). Antiviral activity of overexpressed IFN peptides was assayed as the inhibition of the cytopathic effect (CPE) of PRRSV and VSV on MARC-145 and PK-15 cells, respectively, in which 1 unit is defined as the highest dilution that reduced cell loss by 50% according to the Reed-Muench method [11].

## Results and conclusions

### Existence of multiple polymorphic isoforms in IFN-α, IFN-δ and IFN-ω subtypes

Summarized in Table 1 are 18 polymorphic isoforms that have non-conservative amino acid changes and potentially show altered antiviral activity. Indeed, single nucleotide polymorphisms (SNP) have been found in most porcine IFN subtypes, in particular in *IFN-α*, *IFN-ω* and *IFN-δ* subclasses, which consist of multiple subtypes. Among all SNPs, approximately 50% cause changes at the amino acid level, including non-conservative substitution of certain residues and deletions resulting in frame shift of translation. The SNP sites are detected along all regions including signal peptides and the five helical regions but are concentrated within the middle region of 40-150 amino acid residues.

**Table 1:** Polymorphic isoforms of porcine type I interferons (IFNs)

IFN polymorphic isoforms	Different from reference sequences^1^ NA → AA	Potential responsible mutations^2^
**IFNα2-1**	7 SNP 5 AA	L^40^-F, C^109^-R
**IFNα2-2**	5 SNP 3 AA	A^127^-V
**IFNα3-1**	5 SNP 4 AA	K^145^-E,
**IFNα3-2**	7 SNP 6 AA	L^40^-F
**IFNα6-1**	(Wild type)	
**IFNα6-2**	4 SNP 1 AA	D^101^-N
**IFNα8-1**	8 SNP 5 AA	N^17^-D
**IFNα8-2**	7 SNP 4 AA	
**IFNα8μ**	8 SNP 2 AA	T^9^-M, d(R^46^)
**IFNα10-1**	12 SNP 8 AA	F^88^-S
**IFNα10-2**	12 SNP 8 AA	K^73^-E
**IFNα12-1**	3 SNP	S^158^-N, P^161^-L
**IFNα12-2**	4 SNP 1 AA	L^90^-P, D^101^-N, A^127^-V, V^174^-A
**IFNα12μ**	10 SNP (ORF)	ORF shifted after 100 AA
**IFNδ1-1**	1 SNP 1 AA	T^65^-A
**IFNδ1-2**	2 SNP 1 AA	N^91^-T
**IFNω5-1**	3 SNP 1 AA	C^52^-R
**IFNω5-2**	1 SNP	
**IFNω5μ**	6 SNP (ORF)	d(stop) and added C-17 AA

1.Reference sequences were extracted from genomic draft sequences by Sanger Institute Porcine Project Team at the Wellcome Trust Sanger Institute and can be obtained from http://www.ensembl.org/Sus_scrofa/index.html. NA, Nucleic acid; AA, amino acid; SNP, single nucleotide polymorphism; (ORF), shifted open reading frame. 2. Potential responsible mutations are defined as mutations at the protein levels in the predicted mature peptide region and those occurring only in the isoforms with altered antiviral activity. Amino acid residues are shown as single letter symbols, L^40^-F indicating the 40^th^ leucine mutated to phenylalanine, for example. d(X) or d(stop), deletion of indicated residue X or stop codon.

### Diverse activity of polymorphic type I IFNs

**Figure 1:**
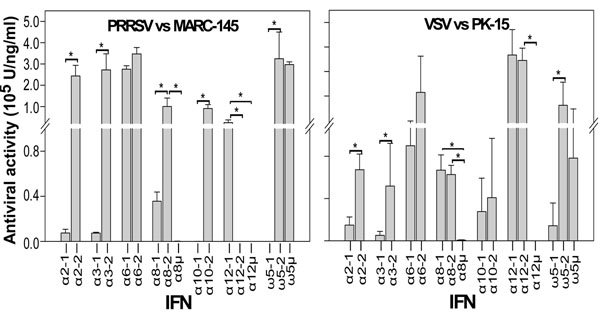
**Differential antiviral activity among polymorphic isoforms of some subtypes of porcine type I IFNs**. Antiviral activity against PRRSV and VSV was evaluated as described in Sang et al., 2010 [11], and analyzed based on individual polymorphic isoforms. Authentic expression of individual IFN peptides was confirmed by gel electrophoresis. Peptide concentrations were adjusted to 2 μg/ml to ensure the antiviral differences were not a result of peptide levels in the antiviral assays. Antiviral activity of overexpressed IFN peptides was assayed as the inhibition of virus cytopathic effect, in which 1 unit (U) is defined as the highest dilution that reduced cell loss by 50% according to the Reed-Muench method. Data are means from four duplicates of two independent experiments. *p<0.05, n=4.

Using IFN polypeptides expressed in a mammalian cell system [11], eight polymorphic IFNs showed altered antiviral activity against both PRRSV and VSV *in vitro* considering the different effects of cytopathic inhibition with the same amount of peptides. In comparison to the wild-type or the variant with more similarity to the wild-type, residual substitution of Leu^40^-Phe and Cys^109^-Arg in IFN-α2-1, Lys^145^-Glu in IFN-α3-1, deletion of Arg^46^ in IFN-α8μ, Phe^88^-Ser in IFN-α10-1, and Cys^52^-Arg in IFN-ω5-1, dramatically reduced antiviral activity against both PRRSV and VSV. The mutations had a more profound effect on reducing anti-PRRSV activity in MARC-145 cells than anti-VSV activity in PK-15 cells (Fig. 1). In addition, deletion mutants, such as IFN-α8μ and IFN-α12μ which are more atypical in their peptide sequence, showed little antiviral activity in both virus-cell systems. Because polymorphism among porcine type I IFN genes may represent an important factor for determining the outcome of the host-virus interaction, it will be informative to correlate IFN polymorphism in pigs with susceptibility to PRRSV and severity of PRRSV infection.

## Competing interests

The authors declare no competing interests related to this manuscript.
